# Epidemiology and pathobiology of H5Nx highly pathogenic avian influenza in South Korea (2003–2024): a comprehensive review

**DOI:** 10.1080/01652176.2025.2498918

**Published:** 2025-05-07

**Authors:** Sun-Hak Lee, Jung-Hoon Kwon, Sungsu Youk, Sang-Won Lee, Dong-Hun Lee, Chang-Seon Song

**Affiliations:** a Avian Disease Laboratory, College of Veterinary Medicine, Konkuk University, Seoul, Republic of Korea; b Wildlife Health Laboratory, College of Veterinary Medicine, Konkuk University, Seoul, Republic of Korea; c Laboratory of Veterinary Microbiology, College of Veterinary Medicine, Kyungpook National University, Daegu, Republic of Korea; d Department of Microbiology, College of Medicine, Chungbuk National University, Cheongju-si, Republic of Korea; e College of Veterinary Medicine, Konkuk University, Seoul, Republic of Korea

**Keywords:** Highly pathogenicity avian influenza, poultry, wild bird, outbreak, republic of Korea, characteristics

## Abstract

Since their emergence in Guangdong, China, in 1996, Gs/GD H5 highly pathogenic avian influenza viruses (HPAIVs) have diversified into multiple clades, spreading globally through wild bird migrations and causing substantial losses in poultry and wildlife. In South Korea, HPAIVs, including H5N1, H5N8, and H5N6 subtypes, have been repeatedly introduced since 2003. This review examines the epidemiology, genetic characteristics, and pathobiological features of these viruses in South Korea. Outbreaks typically occur between October and December, aligning with the arrival of wintering migratory birds. While outbreaks in poultry farms dominated before 2018, wild bird cases became more prevalent in subsequent years. Seasonal outbreaks in poultry have declined, but large-scale mortality events in wild birds emerged biennially from 2020. Genotypic diversity has increased since 2014 due to reassortment with low pathogenic viruses, with novel genomic traits detected in recent seasons. Infection studies show consistently fatal outcomes in chickens, while high mortality in domestic ducks was observed only with two of the studied strains, despite efficient transmission. Wild bird studies reveal species-specific roles in viral shedding and transmission. This review underscores the dynamic nature of HPAI outbreaks, highlighting the importance of surveillance, biosecurity, and genetic and pathogenicity analyses to mitigate future risks.

## 1. Introduction

High pathogenicity avian influenza (HPAI) is an acute and highly infectious viral disease caused by high pathogenicity avian influenza virus (HPAIV), first detected in domestic geese located in the Guangdong province of China in 1996 (Xu et al. 1999). Initially confined to poultry species and Southeast China, the identified HPAIV subtype H5N1 re-emerged in 2003, triggering large-scale poultry outbreaks across several Asian countries (Cauthen et al. 2000; Guan et al. 2002; Li et al. 2010). This HPAIV also transmitted to the migratory waterfowls, spread to neighboring countries, including the Republic of Korea, Japan, Cambodia, Indonesia and Thailand (Alexander 2007).

The evolution of Goose/Guangdong (Gs/GD) HPAIV within clades has demonstrated dynamic changes, particularly in the context of prolonged circulation in poultry. Initially, the prevailing subclades of clade 2 H5 viruses underwent replacement by an antigenically distinct subclade. This shift was followed by the emergence of subclades 2.2 (Nagarajan et al. 2012) and 2.3.2.1 (Creanga et al. 2013). Further evolution within this framework led to the development of three distinct subclades, namely 2.3.2.1a, 2.3.2.1b, and 2.3.2.1c, with a predominant impact observed in China and Southeast Asia (Bi et al. 2016). Since 2008, various HPAI subtypes carrying the genetic foundation of the Gs/GD lineage H5 clade 2.3.4.4 have been identified in domestic ducks, showing ongoing evolutionary adaptations. These subtypes have subsequently diversified into different subclades in domestic avian populations (Lee, Bertran, et al. 2017). Notably, certain subclades, such as 2.3.2.1c H5N1 and 2.3.4.4b H5Nx HPAI, have demonstrated intercontinental spread by wild migratory birds. This phenomenon has resulted in intercontinental waves (waves 2 and 3a) and subsequent waves (waves 3b and 4), signifying the far-reaching consequences of HPAIV evolution in global avian populations (Lee et al. 2021).

South Korea has experienced a recurring influx of 11 HPAIV H5Nx subtypes carrying multiple distinct reassortants, several of which were linked to viruses involved in intercontinental transmission events-such as wave 1, 2, and 4 (Lee et al. 2021). To date, a review, including a comprehensive genetic analysis and pathobiological characteristics of all HPAIV H5Nx viruses occurred in South Korea, has yet to be compiled. This paper aims to assess and present the genetic evolution and molecular epidemiology of all assorted HPAIV H5 outbreaks in South Korea since the first outbreak in 2003 with a temporal manner. Clade and subclade designations follow the WOAH H5 nomenclature system to facilitate international comparison, while the terms ‘genotype’ and ‘subgroup’ are locally defined classification within South Korea; ‘genotype’ refers to the genome constellation based on the eight gene segments, and ‘subgroup’ is defined by phylogenetic clustering of the HA gene. Additionally, this review contains pathobiological investigations for representative HPAIVs in each outbreak in South Korea.

## 2. 2003–2004: Clade 2.5 H5N1

Before 2003, South Korea had not experienced any documented case of HPAI outbreaks. An exception occurred in 2001, when a singular case emerged, linked to the consumption of imported Chinese duck meat (Tumpey et al. 2003). The Hemagglutinin (HA) gene of this isolate exhibited clustering with the H5 Gs/GD lineage. Furthermore, a high level of sequence similarity (98.4%) was observed between these isolated HPAIVs, and both chicken and human isolates from Hong Kong.

**Figure 1.**
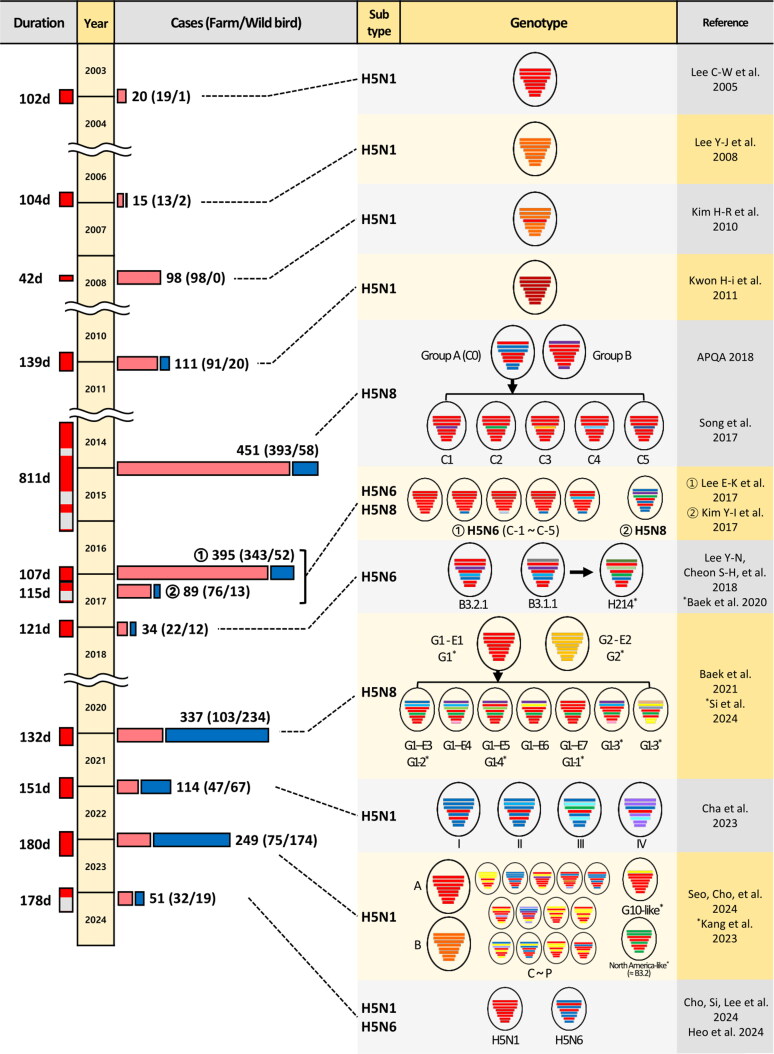
Timeline, case counts, and genotypes of highly pathogenic avian influenza (HPAI) outbreaks in South Korea from 2003 to 2023. The first three columns show duration and case counts of all HPAI H5 reports in South Korea, sourced from KAHIS (home.kahis.go.kr) and WADIS (wadis.go.kr), provided by the animal and plant quarantine agency and the wildlife disease control center, respectively. The leftmost column indicates the outbreak duration; ‘d’ stands for days. Outbreak periods are highlighted in red, marking the time between the first and last cases in each outbreak. Outbreaks are segmented if there is a gap of over four months (120 days) since the previous case, with the 2016–2017 period further divided by subtype. Case counts are represented by pink bars (poultry cases) and blue bars (wild bird cases). The two right columns display confirmed genotypes and reference studies corresponding to each outbreak period. Genotype names are presented as designated in each original reference. Bars inside black circles represent the eight gene segments of the virus, ordered from top to bottom as follows: polymerase basic 2, polymerase basic 1, polymerase acidic, hemagglutinin, nucleoprotein, neuraminidase, matrix, and nonstructural. Different bar colors indicate viral origins inferred from maximum-likelihood phylogenetic trees, according to the references in the fourth column. Black arrows indicate the direction of genetic contribution from donor viruses to newly emerged recombinant viruses. Asterisks indicate entries with different references, placed before the reference and after the genotype name.

On December 10, 2003, the first occurrence of HPAI was reported in South Korea. There were reported outbreaks on poultry farms, including duck farms (Kwon, Joh et al. 2005), and broiler breeder farms (Kwon, Sung, et al. 2005). In wild birds, the virus was isolated from three magpies (*Pica pica*) in Yangju, Gyeonggi Province, in March 2004, where the first farm case of HPAI was reported (Kwon et al. 2005). The initial isolates from duck farms were subjected to pathogenicity experiments on SPF chickens, and the symptoms observed on the farms were reproduced (Kwon, Joh, et al. 2005). The reported H5N1 strains in Asia, including the Korean isolates, shared a gene constellation similar to that of the Hong Kong isolates from late 2002 and contained some molecular markers that seem to have been fixed in the Gs/Gd lineage virus since 2001, such as amino acid deletion in the NA stalk region (49–68) and the NS1 protein (80–84) (Figure 1) (*Lee et al. 2005).* However, the topology of the phylogenetic tree clearly differentiates the Korean isolates from the Vietnamese and Thai isolates (Lee *et al.*^2005^). For this period, 19 farm cases and one wild bird case were reported (Figure 1 and Table 1).

**Table 1.** Case counts of all HPAIV reports in South Korea retrieved from the epidemiological investigation report (home.kahis.go.kr, http://wadis.go.kr).

Period	‘03/04	‘06/07	‘08	‘10/11	‘14/16	‘16/17	‘17	‘17/18	‘20/21	‘21/22	‘22/23	‘23/24
Subtype	H5N1	H5N1	H5N1	H5N1	H5N8	H5N6	H5N8	H5N6	H5N8	H5N1	H5N1	H5N1/H5N6
Clade	2.5	2.2	2.3.2	2.3.2	2.3.4.4	2.3.4.4e	2.3.4.4b	2.3.4.4b	2.3.4.4b	2.3.4.4b	2.3.4.4b	2.3.4.4b
First report	Dec 2003	Nov 2006	Apr 2008	Dec 2010	Jan 2014	Nov 2016	Fab 2017	Nov 2017	Nov 2020	Nov 2021	Oct 2022	Nov 2023
Duration^a^ (days)	102	104	42	139	811	107	115	121	132	151	180	178
**Farm case** ^b^	**19**	**13**	**98**	**91**	**393**	**343**	**76**	**22**	**103**	**47**	**75**	**32**
A. chicken	10	5	79	34	84	197	52	8	55	22	34	17
B. duck	9	6	18	54	290	136	23	14	48	23	38	15
C. minor poultry	0	2	1	3	19	10	1	0	0	2	3	0
**Wild bird case** ^c^	**1**	**2**	**0**	**20**	**58**	**52**	**13**	**12**	**234**	**67**	**174**	**19**
D. feces	0	2	0	5	17	14	4	10	34	21	31	2
E. captured bird	0	0	0	1	12	0	0	1	19	7	11	5
F. carcasses	1	0	0	14	29	38	9	1	181	39	132	12

^a^ Durations were computed from the first case to the last case in each outbreak. Outbreaks are segmented if there is a gap of over four months (120 days) since the previous case, with the 2016–2017 period further divided by subtype.

^b^ Farm cases include outbreaks in domestic chickens (A), domestic ducks (B), and minor poultries (C), such as quails and pheasants.

^c^ Wild bird cases were categorized based on sample types, including feces (D), captured birds (E), and carcasses (F).

Animal experiments were conducted on four types of poultry using viruses circulated in this period: White Plymouth Rock and White Leghorn (WL) chickens (*Gallus gallus domesticus*), Japanese quail (*Coturnix coturnix japonica*), and Pekin ducks (*Anas platyrhyncos domesticus*) (Lee et al. 2005) (Table 2). The farm-origin HPAIVs, CK/Korea/ES/03 and DK/Korea/ES1/03, caused systemic infections in chickens and quail, resulting in the death of all birds within 2 and 4 days of intranasal inoculation, respectively. This isolate also replicated in multiple organs of ducks and caused some mortality. For a study using wild birds species, high susceptibility of magpies to HPAIV was observed (Table 3), consistent with the case report describing magpies mortality (Kwon, Joh, et al. 2010).

**Table 2.** Experimental infections in SPF chickens and various species of domestic birds with HPAIVs isolated in South Korea from 2003 to 2022.

period	H5 Clade^a^	sub-type	Strain	animal	pathogenicity index	contents	results		reference
‘03-04	2.5	H5N1	CK/Korea/ES/03	6-week-old SPF^b^ chicken		Pathogenicity	(i.v.) Mortality (8/8)	Tissue distribution^k^ (Brain, heart, lung, trachea, cloaca)	Kwon, Joh, et al. (2005)
				4-week-old SPF White Plymouth Rock	(i.v.) MDT^c^ = 1.0 (i.n.) MDT = 1.9	Pathogenicity Histopathology Tissue viral titers	(i.v.) Mortality (8/8) Mortality (8/8)	Tissue distribution (Brain, heart, lung, trachea, cloaca)	Lee et al. (2005)
				4-week-old White Leghorn chickens	(i.n.) MDT = 2.0		Mortality (8/8)	Tissue distribution (Brain, heart, lung, trachea, cloaca)	
				5-week-old Japanese quail	(i.n.) MDT = 3.8		Mortality (7/7)	Tissue distribution (Brain, heart, lung, trachea, cloaca)	
				2-week-old Pekin ducks	(i.n.) MDT = 4.0		Mortality (2/8)	Tissue distribution (Brain, heart, lung, trachea, cloaca)	
				2-week-old Pekin		Transmissibility	(i.v.) Transmission^j^ (9/10) Transmission (4/10)		
‘06-07	2.2	H5N1	A/EM/Korea/W149/06	5-week-old SPF White leg-horn chicken	cLD_50_^d^ = 10^2.3^EID_50_^e^	Pathogenicity Transmission Viral shedding Tissue viral titer	Mortality (7/7) Transmission (3/3)	Virus shedding^k^ OP 7/7, CL 7/7 Tissue distribution (Lung, Brain, Kidney, Spleen, Colon, Heart)	Kwon et al. (2011)
				3-week-old domestic duck	dLD_50_^f^<10^2.0^EID_50_		Mortality (7/7) Transmission (3/3)	Virus shedding OP 7/7, CL 7/7 Tissue distribution (Lung, Brain, Kidney, Spleen, Colon, Heart)	
‘08	2.3.2	H5N1	Ck/Kr/Gimje/08	2-week-old domestic duck	MDT = 3d	Pathogenicity Tissue viral titer Viral shedding Tissue viral titer	Mortality (8/8)	Virus shedding OP 8/8, CL 8/8 Tissue distribution (Brain, trachea, lung, kidney, spleen, heart, cecal tonsil, liver)	Kang H-M et al. 2013
‘10-11	2.3.2	H5N1	A/Md/Korea/W401/11	5-week-old SPF White leg-horn chicken	cLD_50_ = 10^3.0^EID_50_	Pathogenicity Transmission Viral shedding Tissue viral titer	Mortality (5/5) Transmission (3/3)	Virus shedding OP 7/7, CL 7/7 Tissue distribution (Lung, Brain, Kidney, Spleen, Colon, Heart)	Kwon et al. (2011)
				3-week-old domestic duck	dLD_50_ = 10^5.3^EID_50_		Mortality (5/5) Transmission (3/3)	Virus shedding OP 7/7, CL 7/7 Tissue distribution (Lung, Brain, Kidney, Spleen, Colon, Heart)	
			A/duck/Korea/Cheonan/2010	chicken	IVPI^g^ = 3.0	Pathogenicity			Kim H-R et al. 2012
	2.3.2.1c	H5N1	A/mandarin duck/Korea/PSC24-24/2010	6-week-old chicken	IVPI = 2.74 MDT = 58.2 h (i.n.) MDT = 75 h	Viral shedding Survival curve Transmission Tissue viral titer			Choi et al. 2013
				2-week-old duck			Mortality (3/6) Transmission (3/3)	Virus shedding OP 8/8, CL 8/8 Tissue distribution (Brain, trachea, lung, spleen, kidney, heart, muscle, cecal tonsil)	
	2.3.2.1c	H5N1	A/Eurasian eagle owl/Korea/23/2010	6-week-old chicken	IVPI = 2.86 MDT = 44.1 h			Tissue distribution (Brain, trachea, lung, spleen, kidney, heart, muscle, cecal tonsil)	
				2-week-old duck			Mortality (6/6) Transmission (2/2)	Virus shedding OP 8/8, CL 8/8 Tissue distribution (Brain, trachea, lung, spleen, kidney, heart, muscle, cecal tonsil)	
‘14-16	2.3.4.4c	H5N8	A/broiler duck/Korea/H1731/2014	4-week-old SPF white leghorn chicken	cLD_50_ = 10^4.4^EID_50_	Transmission Viral shedding Tissue tropism	Mortality (5/5) Transmission (1/3)	Virus shedding OP 5/5, CL 5/5 Tissue distribution (brain, heart, kidney, lung cecal tonsil, pancreas, proventriculus, spleen, muscle, liver, thymus, trachea)	Baek et al. 2022
			A/domestic mallard duck/Korea/ H1924/2014		cLD_50_ = 10^4.5^EID_50_		Mortality (5/5) Transmission (3/3)		
			A/mallard duck/Korea/H2102/2015		cLD_50_ = 10^3.5^EID_50_		Mortality (5/5) Transmission (3/3)		
	2.3.4.4c	H5N8	A/breeder duck/korea/Gochang1/2014	5-week-old SPF chicken	IVPI = 3.0 MDT = 2.5d	Pathogenicity Transmission Viral shedding Serology Tissue tropism	Mortality (8/8) Transmission (1/3)	Virus shedding OP 8/8, CL 8/8 Tissue distribution (Trachea, lung, liver, spleen, kidney, cecal tonsil, proventriculus, Intestine (pancreas), heart, muscle, brain)	Song et al. 2015
	2.3.4.4b		A/broiler duck/korea/Buan2/2014		IVPI = 3.0 MDT = 4.5		Mortality (8/8) Transmission (2/3)		
	2.3.4.4c	H5N8	A/breeder duck/Kr/Gochang1/2014	2-week-old Pekin ducks		Viral shedding Transmission Tissue viral titer	Mortality (1/5) Transmission (1/3)	Virus shedding OP 9/9, CL 9/9	Kang H-M et al. 2015
			A/Baikal teal/Kr/Donglim3/2014	2-week-old Pekin ducks			Mortality (1/5) Transmission (0/3)	Virus shedding OP 9/9, CL 9/9	
	2.3.4.4b		A/broiler duck/Kr/Buan2/2014	2-week-old Pekin ducks			Mortality (0/5) Transmission (1/3)	Virus shedding OP 9/9, CL 9/9	
	2.3.4.4c	H5N8	MDk/W452	3-week-old SPF chicken		Pathogenicity Transmission	Mortality (6/6)	Transmission (3/6)	Bae et al. 2015
				3-week-old commercial layer			Mortality (6/6)	Transmission (3/6)	
		H5N8	A/Baikal teal/Korea/K14-E016/2014	3-week-old commercial layer chickens	MDT = 4.8d	Transmission Pathogenicity Viral shedding	Mortality (8/8) Transmission (1/6)	Virus shedding OP 8/8, CL 8/8	Lee DH et al. 2016
				3-week-old commercial quails	MDT = 2d		Mortality (4/4) Transmission (2/4)	Virus shedding OP 4/4, CL 4/4	
				10-week-old Korean native chickens			Mortality (3/5) Transmission (1/3)	Virus shedding OP 4/5, CL 3/5	
				8-week-old muscovy ducks			Mortality (0/4) Transmission (4/4)	Virus shedding OP 4/4, CL 4/4	
	2.3.4.4b	H5N8	A/broiler duck/korea/Buan2/2014	5-week-old SPF White leg-horn chicken	cLD_50_=10^5.3^EID_50_ MDT = 3.2d	Pathogenicity Tissue viral titer	Mortality (5/5)	Tissue distribution (Trachea, heart, lung, kidney, brain, pancreas, cecal tonsil, liver, spleen, muscle, proventriculus)	Lee E-K et al. 2016
				5-week-old Korean native chicken	cLD_50_ = 10^6.7^EID_50_ MDT = 4.4d		Mortality (5/5)	Tissue distribution (Trachea, heart, lung, kidney, brain, pancreas, cecal tonsil, liver, muscle, proventriculus)	
‘16-17	2.3.4.4b	H5N8	CT/W555/H5N8	chicken	IVPI = 2.94 MDT = 36h	Pathogenicity	(i.v.) Mortality (10/10)		Kim et al. 2017
	2.3.4.4e	H5N6	A/duck/Korea/Es2/2016	6-week-old SPF white leghorn chicken	cLD_50_ = 10^3.7^ EID_50_ MDT = 2.6d IVPI = 3.0	Pathogenicity Transmission Histopathology Viral shedding	Mortality (8/8) Transmission (3/3)	Virus shedding OP 8/8, CL 8/8	Park et al. 2019
				6-week-old broiler chicken (white line)	BLD_50_^h^=10^3.8^ EID_50_ MDT = 2.1d		Mortality (8/8) Transmission (3/3)	Virus shedding OP 8/8, CL 8/8	
				8-week-old KNC (brown line)	BLD_50_=10^4.3^ EID_50_ MDT = 3.1d		Mortality (5/5) Transmission (3/3)	Virus shedding OP 8/8, CL 8/8	
‘16-17	2.3.4.4e	H5N6	A/Mandarin_duck/Korea/K16-187-3/2016	2-week-old domestic ducks	BID_50_^i^=10^3.0^ EID_50_ dLD_50_>10^6.0^ EID_50_ MDT = 3.5d	Pathogenicity Histopathology Viral shedding	Mortality (2/5) Transmission (2/3)	Virus shedding OP 7/7, CL 7/7	Kwon J. H. et al. 2019
			A/duck/Korea/Es2/2016		BID_50_ = 10^3.0^ EID_50_ dLD_50_ = 10^4.0^EID_50_ MDT = 7.5d		Mortality (4/5) Transmission (3/3)	Virus shedding OP 7/7, CL 7/7	
‘17-18	2.3.4.4b	H5N6	A/duck/Korea/HD1/2017	5-week-old SPF chicken	IVPI = 2.98 cLD_50_ = 10^3.6^EID_50_ MDT = 2.2d	Pathogenicity Transmission Tissue viral titer Viral shedding	Mortality (5/5) Transmission (1/2)	Virus shedding OP 5/5, CL 5/5 Tissue distribution (Trachea, thymus, liver, heart, cecal tonsil, proventriculus, muscle lung, kidney brain, spleen, pancreas)	Park M-J et al. 2021
				2-week-old duck	BID_50_=10^5.0^EID_50_		Mortality (0/5) Transmission (0/3)	Virus shedding OP 5/5, CL 5/5 Tissue distribution (Trachea, thymus, proventriculus, pancreas)	
‘20-21	2.3.4.4b	H5N8	A/mandarin duck/Korea/H242/2020	5-week-old SPF chicken	IVPI = 2.88 cLD_50_ = 10^4.5^ EID_50_ MDT = 4.3d	Pathogenicity Transmission Viral shedding	Mortality (5/5) Transmission (1/3)	Virus shedding OP 5/5, CL 5/5 Tissue distribution (Trachea, thymus, liver, heart, cecal tonsil, proventriculus, muscle lung, kidney brain, spleen, pancreas)	Park M-J et al. 2021
				2-week-old duck	BID_50_=10^5.3^EID_50_		Mortality (0/5) Transmission (0/3)	Virus shedding OP 4/5, CL 4/5 Tissue distribution (Trachea, thymus, liver, heart, cecal tonsil, proventriculus, muscle, lung, kidney, spleen)	
‘21-22	2.3.4.4b	H5N1	A/mandarin duck/Korea/WA585/2021	5-week-old SPF chicken	IVPI = 2.98 MDT = 2.6d cLD_50_=10^3.7^EID_50_	Pathogenicity Tissue viral titer Viral shedding Serology	Mortality (5/5) Transmission (3/3)	Virus shedding OP 5/5, CL 5/5 Tissue distribution (Trachea, thymus, liver, heart, cecal tonsil, proventriculus, muscle, lung, kidney, brain, spleen, pancreas)	Cha et al. 2023
				2-week-old duck	BID_50_ = 10^3.2^EID_50_		Mortality (0/5) Transmission (0/3)	Virus shedding OP 5/5, CL 5/5 Tissue distribution (Trachea, thymus, liver, heart, cecal tonsil, proventriculus, lung, kidney, brain, spleen, pancreas)	

^a^ Lineage of virus used in each study was replaced with updated unified nomenclature made by WOAH.

^b^ SPF: Specific-Pathogen-Free.

^c^ MDT: mean death time.

^d^ cLD_50_: 50% chicken lethal dose.

^e^ EID_50_: 50% egg infectious dose.

^f^ dLD_50_: 50% duck lethal dose.

^g^ IVPI: Intravenous pathogenicity index.

^h^ BLD_50_: 50% bird lethal dose.

^i^ BID_50_: 50% bird infectious dose.

^j^ Transmission, mortality based on highest viral challenge groups.

^k^ Virus shedding and tissue distribution deduced by viral detection. Virus shedding only stated for inoculated birds. OP: oropharyngeal swab; CL: cloacal swab.

**Table 3.** Experimental infections in various species of wild birds with HPAIVs isolated in South Korea from 2003 to 2022.

Period	H5 Clade^a^	sub-type	Strain	Animal	Pathogenicity index	Contents	Results		Reference
‘03–04	2.5	H5N1	CK/Korea/ES/03	wild magpie	MDT^b^ = 5.8d	Histopathology	Mortality^c^ (9/9)		Kwon, Joh et al. (2010)
‘06–07	2.2	H5N1	A/chicken/South Korea/IS/06	mute swan		Pathogenicity Viral shedding Transmissibility Serology	Mortality (2/2) Transmission^c^ (1/1)	Virus shedding^d^ OP 2/2, CL 2/2 Tissue distribution (Nasal cavity, lung, heart, vessel, brain, nerve, spleen, liver, pancreas)	Kwon, Thomas, et al. (2010)
				ruddy shelduck			Mortality (2/2) Transmission (1/1)	Virus shedding OP 2/2, CL 0/2 Tissue distribution (Nasal cavity, lung, heart, brain, nerve, pancreas)	
				Greylag goose			Mortality (0/2) Transmission (1/1)	Virus shedding OP 2/2, CL 1/2 Tissue distribution (Brain)	
				mandarin duck			Mortality (0/2) Transmission (1/1)	Virus shedding OP 2/2, CL 2/2 Tissue distribution (Nasal cavity, brain, pancreas)	
				mallard			Mortality (0/2) Transmission (0/1)	Virus shedding OP 2/2, CL ½	
			A/chicken/Kr/IS/2006	adult mandarin duck		Pathogenicity Transmission Viral shedding Tissue viral titer	Mortality (0/5) Transmission (2/2)	Virus shedding OP 5/5, CL 5/5 Tissue distribution (Trachea, Cecal tonsil, Lung)	Kang, Lee, et al. (2017)
‘10-11	2.3.2.1c	H5N1	A/mandarin_duck/Kr/PSC24-24/2010	adult mandarin duck		Pathogenicity Transmission Tissue viral titer Viral shedding	Mortality (0/5) Transmission (2/2)	Virus shedding OP 5/5, CL 5/5 Tissue distribution (Lung)	Kang, Lee, et al. (2017)
	2.3.2.1	H5N1	A/mandarin duck/Korea/K10-483/ 2010	northern pintail		Transmission Viral shedding	Mortality (0/3) Transmission (direct 0/3, indirect 0/2)	Virus shedding OP 2/3, CL 0/3	Kwon et al. (2018)
‘14-16	2.3.4.4b	H5N8	A/broiler duck/Kr/Buan2/2014	Baikal teal		Viral shedding Transmission Tissue viral titer		Virus shedding OP 1/2, CL 1/2	Kang et al. (2015)
		H5N8	A/broiler_duck/Kr/Buan2/2014	adult mandarin duck		Pathogenicity Transmission Tissue viral titer Viral shedding	Mortality (0/5) Transmission (2/2)	Virus shedding OP 5/5, CL 5/5 Tissue distribution (Trachea, proventriculus, pancreas, cecal tonsil, lung, heart)	Kang et al. (2017)
	2.3.4.4c	H5N8	A/baikal teal/Korea/2406/2014	mandarin duck		Pathogenicity Transmission	Mortality (0/4)	Transmission (2/2)	Kwon, Noh, et al. (2017)
				domestic pigeon			Mortality (0/5)	Transmission (1/3)	
			A/Mallard/Korea/KU3-2/2015	mandarin duck			Mortality (0/4)	Transmission (1/2)	
				domestic pigeon			Mortality (0/3)	Transmission (0/3)	
		H5N8	A/mallard/Korea/KU3-2/2015	northern pintail		Transmission Viral shedding	Mortality (0/3) Transmission (direct 2/3, indirect 2/2)	Virus shedding OP 3/3, CL 2/3	Kwon et al. (2018)
‘16-17	2.3.4.4	H5N6	A/Whooper _swan/Korea/Gangjin/ W49_1/2016	mandarin duck		Pathogenicity Transmission Tissue viral titer Viral shedding	Mortality (0/3) Transmission (3/3)	Virus shedding OP 3/3, CL 3/3 Tissue distribution (Trachea, Lung, Kidney, Spleen, cecal tonsil, Liver, Muscle, Intestine, Pancreas, Proventriculus)	Son et al. (2018)
	2.3.4.4b	H5N8	A/Grey heron/Korea/W779/2017				Mortality (0/3) Transmission (3/3)	Virus shedding OP 3/3, CL 3/3 Tissue distribution (Trachea, Lung, Spleen, cecal tonsil, Intestine, Proventriculus)	

^a^ Lineage of virus used in each study was replaced with updated unified nomenclature made by WOAH.

^b^ MDT: mean death time.

^c^ Transmission, mortality based on highest viral challenge groups.

^d^ Virus shedding and tissue distribution deduced by viral detection. Virus shedding only stated for inoculated birds. OP: oropharyngeal swab; CL: cloacal swab.

## 3. 2006–2007: Clade 2.2 H5N1

Following the initial report of a HPAI outbreak in November 2006, the epidemic persisted for 104 days, resulting in 13 confirmed cases on farms and two positive environmental samples (Table 1). Phylogenetic analysis indicated an independent introduction of the Gs/GD-lineage H5 virus, distinct from the strain responsible for the 2003 outbreak (Lee, Choi, et al. 2008). The wild-bird-origin virus A/bar-headed goose/Qinghai/5/2005 was identified as the most closely related strain. Among the affected sites, five farms including three chicken farms, one quail farm, and one duck farm were described in detail, along with two environmental detections (Lee et al. 2008). Additionally, experimental inoculations conducted during that period evaluated the pathogenicity of the isolated virus in five wild bird species: mute swans (*Cygnus olor*), graylag geese (*Anser anser*), ruddy shelducks (*Tadorna ferruginea*), mallards (*Anas platyrhynchos*), and mandarin ducks (*Aix galericulata*) (Kwon, Thomas, et al. 2010) (Table 3).

## 4. 2008: Clade 2.3.2 H5N1

The initial report in April 2008 included an analysis of eight genomic segments from six H5N1 strains isolated on early farms outbreaks (Kim et al. 2010). Phylogenetic analysis revealed that the HA gene clustered with clade 2.3.2 viruses, while the internal and neuraminidase (NA) genes were closely related to clade 2.3.4 viruses (Figure 1). A systematic comparison of pathological lesions in the cases reported from chicken and duck farms that year revealed different pathogenicity patterns compared to the previous year (Woo et al. 2011). The observed increase in mortality among domestic ducks, as compared to previous HPAIV strains, may be attributable to heightened viral cardiotropism, which induces heart failure (Woo et al. 2011). This season, the first outbreak of HPAI in captive birds at a public exhibit was reported, involving two pheasants sourced from a live bird market, four days before their deaths; the market also housed poultry linked to an outbreak farm ­confirmed 6 days later (Yoon et al. 2010). In addition, animal experiments were conducted on domestic ducks and mice for the HPAIV isolated in 2008 (Kang et al. 2013) (Table 2, Supplementary Table 1). In domestic ducks, the virus demonstrated systemic replication across all tissues, leading to 100% mortality accompanied by severe neurological symptoms. In murine models, the infection resulted in a 22.3% reduction in body weight and systemic replication, with a 50% mouse lethal dose of 10^2.2^ 50% egg infectious dose (EID_50_).

## 5. 2010–2011: Clade 2.3.2.1 H5N1

Between December 2010 and May 2011, there were 91 reported HPAI outbreaks in poultry farms and 20 cases detected in wild birds (Figure 1 and Table 1), with several published reports characterizing the HPAIVs isolated from wild birds. Fourteen H5N1 HPAIVs, primarily from Mandarin ducks, were isolated from 728 wild bird fecal samples, formed a cluster with clade 2.3.2 H5 HPAIVs closely related to wild bird-origin strains from Mongolia, based on HA gene analysis (Lee et al. 2011). Additionally, there were reports of H5N1 HPAIV isolation from healthy captured mallards (Kim et al. 2011) and fecal samples of mandarin ducks, as well as carcasses of Eurasian eagle owls (*Bubo bubo*) (Choi et al. 2013). Two strains isolated from mallard fecal samples in early 2011 showed the highest sequence similarities to the 2011 Japanese strain A/Ws/Hokkaido/4/11 and 2009–2010 Mongolian-like clade 2.3.2 isolates, differing from previous Korean H5N1 viruses (Kwon et al. 2011).

During the outbreak period, Rhyoo et al. conducted a comparative analysis of clinical signs, histopathological lesions, and viral antigen distribution in naturally infected meat-type ducks and breeder ducks (Rhyoo et al. 2015). Commercial meat-type ducks exhibited higher virus titers in organs, increased virus shedding, and higher mortality rates, while breeder ducks only showed decreased egg productions. Genetic analysis indicated that the isolates were distinct from strains isolated in 2003, 2006, and 2008, displaying high similarity (>99%) to H5N1 HPAIVs detected in Mongolia, China, and Russia in 2009–2010 (Kim et al. 2012) (Figure 1).

In animal experiments, pathogenicity studies were conducted on Mandarin duck (Kang et al. 2017) and Northern pintail (Kwon, Le, et al. 2018) (Table 3). Mandarin duck exhibited low pathogenicity, with no observed mortality but confirmed shedding and direct contact transmission, whereas Northern pintails showed neither clinical signs nor mortality. In infection experiments, high pathogenicity potential was evident in chickens and mice, contrasting with ducks and ferrets, demonstrated low pathogenicity (Kwon et al. 2011) (Table 2, Supplementary Table 1). Using viruses isolated from Mandarin duck and Eurasian eagle owls during the same season, experimental inoculation revealed high pathogenicity in chickens and ducks, along with high pathogenicity in mice, even in the absence of prior viral adaptation (Choi et al. 2013) (Table 3, Supplementary Table 1).

## 6. 2014–2016: Clade 2.3.4.4 H5N8

In 2014, the introduced virus was identified as the H5N8 subtype, marking the first shift in the NA subtype. This outbreak lasted a record 811 days, from January 2014 to April 2016, the longest duration documented to date. On January 16, 2014, a breeder duck farm near Donglim Reservoir in Jeonbuk Province firstly reported a decrease in egg production and a slight increase in mortality rates among ducks (Lee et al. 2014), followed by a large-scale of outbreaks in poultry farms. Regarding cases in local broiler breeder farms and a commercial layer farm, a gradual increase in mortality, slow transmission, and unrecognizable clinical signs of HPAI were observed (Bae et al. 2015).

During this season, 393 farm cases and 58 wild bird cases were reported (Table 1). Particularly, mass die-off of Baikal teals was observed at Donglim Reservoir, with 19 confirmed cases of HPAI (Kim. Kwon, et al. 2015). Gross and histologic lesions were observed in the ­pancreas, lung, brain, and kidney of Baikal teals (*Anas formosa*), bean geese (*Anser fabalis*), and whooper swans (*Cygnus cygnus*), but not in mallard ducks (Kim, Kwon, et al. 2015). In February 2014, HPAI was also reported in the feces of wild mallard ducks in urban areas, representing a novel occurrence (Kwon, Lee, Jeong, et al. 2017). A month later, the HPAI H5N8 virus was isolated from a reared ostrich in a zoo (Kim et al. 2016). From December 2014 to February 2015, surveillance of wild bird feces (980 samples) and swab samples (102 samples) resulted in the isolation of 11 H5N8 viruses bearing a different cluster from an icA3 cluster (Lee et al. 2015), designated to the C2 subgroup (Song et al. 2017), from the viruses previously reported (Kwon et al. 2016). Full genome sequencing of 37 HPAIVs isolated from wild birds and poultry revealed two genotypes (A and B) (Figure 1) (APQA 2018). The virus was introduced through wild bird migration, and only the predominant genotype A continued to circulate (Jeong et al. 2014). Genetic analyses of 101 H5N8 viruses that occurred in South Korea from 2014 to 2016 revealed that the primitive H5N8 (C0, A) formed multiple HA subgroups (C1-C5) along with the viruses circulating on poultry farms (Song et al. 2017) (Figure 1).

The pathogenicity of the H5N8 virus from this ­season was evaluated in mice, ferrets, chickens, ducks, dogs, and cats (Kim et al. 2014) (Table 2, Supplementary Table 1). The virus demonstrated a high level of pathogenicity in chickens, while in ducks, it exhibited low pathogenicity but high infectivity. Notably, the virus replicated in human respiratory tract tissues, with observed binding to human virus-like receptors. In mice, the virus displayed moderate pathogenicity and limited tissue tropism, while ferrets exhibited a moderate nasal wash titer. Differential pathogenicity among subgroup C1, C2, and C4 viruses in chickens was investigated (Baek et al. 2022) (Table 2, Supplementary Table 1). C1 presented a longer mean death time (MDT), significantly lower viral titers in tissues, and lower transmissibility in chickens compared to C2 and C4. Viral titers were also lower in chickens inoculated with C1 compared to an index H5N8 virus (buan2), suggesting lower adaptation and transmissibility in the C1 subgroup. Pathological analysis of HPAIV, belongs to the C4 subgroup, isolated in layer farms was conducted in SPF chickens and commercial layer chickens (Bae et al. 2015) (Table 2). The inoculation group exhibited mortality rates ranging from 50% to 100%, while the contact-exposed group showed delayed or no mortality, irrespective of the chicken type. Intravenous pathogenicity indices of two H5N8 genotypes (A/Breeder duck/Korea/Gochang1/2014, C5 subgroup and A/Broiler duck/Korea/Buan2/2014, C0 genotype) isolated in 2014 were both confirmed as 3.0 in SPF chickens (Song et al. 2015) (Table 2). Contact transmission experiments following intranasal inoculation with the same virus resulted in a 100% mortality rate in the challenged group, with MDTs of 2.5 and 4.5 days. The transmission group exhibited mortality rates of 33.3% and 66.6%. Animal experiments were conducted to assess the pathogenicity of the virus in this season, using commercial layer chickens, quails, Korean native chickens (KNC), and Muscovy ducks (*Cairina moschata*) (Lee, Kwon, et al. 2016) (Table 2). Intranasal inoculation with 10^6.0^ EID_50_ of the virus resulted in 100% mortality in layer chickens (8/8) and quails (4/4), a 60% mortality rate in KNC (3/5), and no deaths in Muscovy ducks (0/4). Virus infection was confirmed in all contact groups. In the case of laboratory verification using the buan2 virus in SPF chickens and KNCs (Lee, Song, et al. 2016), the relative lower pathogenicity in KNCs than SPF chickens, as indicated by a longer MDT and lower viral titers in tissues (Table 2).

For animal experiments using wild birds species (Table 3), experiments involving mallards, Baikal teals, and domestic ducks revealed the moderate pathogenicity of H5N8 viruses, with mortality rates ranging from 0% to 20% (Kang et al. 2015). In wild mallards, both H5N8 and H5N1 viruses did not induce severe illness or death. Viral replication and shedding were higher in H5N8-infected mallards compared to H5N1-infected mallards. Investigations into the pathogenicity and transmissibility of the H5N8 HPAIV isolated from Mandarin duck in 2014 revealed no clinical symptoms or mortality, yet contact transmission occurred, accompanied by higher viral shedding compared to H5N1 subtype HPAI (Kang et al. 2017). Experiments conducted in Mandarin ducks and pigeons utilizing viruses from Baikal teal carcasses in 2014 and mallard feces in 2015 assessed pathogenicity, infectivity, and transmissibility (Kwon, Noh, et al. 2017). No clinical symptoms or mortality were observed, with virus transmission confirmed only in mandarin ducks. Results from animal experiments in Mandarin duck indicated superior replication of the H5N8 virus in the respiratory tracts compared to clade 2.2 and 2.3.2.1c. Examination in Northern pintails showed no clinical symptoms or mortality but increased viral shedding and superior transmission compared to clade 2.3.4.4c virus (Kwon, Lee, et al. 2018).

Intranasal inoculation experiments in dogs with the HPAI H5N8 virus isolated from Baikal teal in 2014 (Yuk et al. 2017) showed no disease, confirming shedding and seroconversion (Supplementary Table 1). Mouse and ferret experiments with H5N8 HPAIVs from C1 to C5 subgroups indicated high pathogenicity in mice, but no clinical symptoms, suggesting poor adaptation to mammals (Lee, Lee, Song, et al. 2018) (Supplementary Table 1). Ferret experiments conducted in 2014 to explore mammalian adaptation and pathogenicity of H5N8 showed no mortality or respiratory symptoms upon intranasal inoculation with the two viruses (Kim et al. 2015) (Supplementary Table 1).

## 7. 2016–2017: Clade 2.3.4.4 H5N6/H5N8

In October 2016, the first detection of H5N6 HPAIV was reported from Mandarin duck feces, identified as a novel reassortant (Kwon, Lee, Swayne, et al. 2017). The PB1 gene closely resembled H4N2 Low Pahogenicity Avain Influenza (LPAI) virus from Guangdong, while other genes shared genetic similarities with H5N6 subtype viruses from China, Vietnam, Laos, and Hong Kong, including human isolates (Figure 1). Two novel H5N6 strains from three whooper swans on November 20, 2016, were mostly related to A/duck/Guangdong/01.01SZSGXJK005-Y/2016 (H5N6) (98.90 ∼ 99.74%), except for the polymerase acidic (PA) gene, showing high ­similarity (99.16%) to an H1N1 LPAI virus from a hooded crane (*Grus monacha*) in Korea in 2016, indicating recombination (Jeong et al. 2017) (Figure 1). For the cases of poultry farms, 343 HPAI outbreaks were reported during 107 days from November 2016 to March 2017, while 52 HPAI cases were reported from wild birds (Figure 1 and Table 1). During this period, reports of mammalian infection emerged, with confirmed HPAI in three cats showing neurological signs near a chicken farm in December 2016 (Lee, Lee et al. 2018). During this period, the identified genotypes of H5N6 HPAIVs, designated as C-1 to C-5 (Jeong et al. 2017; Lee, Song, et al. 2017), were found to result from reassortment with Eurasian H1N1 and H7N7 LPAI viruses, specifically involving the PA and NS genes (Figure 1).

On January 27, 2017, a clade 2.3.4.4 H5N8 HPAIV in a dead egret in Jeonju revealed reassortment with HPAIVs from Qinghai Lake and western Siberia, and LPAI viruses from Eurasia (Woo et al. 2017). Die-offs in wild birds occurred in Chungcheongbuk-do in November 2016, with analysis of four H5N6 HPAI isolates from mallard feces showing evidence of recombination with at least three subtypes (H5N6, H4N2, H1N1) (Si et al. 2017) (Figure 1). In December 2016 and January 2017, a H5N8 HPAIV from Common teal (*Anas crecca*) feces in Gyeonggi-do was originated from A/Brk/Korea/Gochang1/14 (H5N8), a minor lineage of H5N8 that emerged in 2014 and disappeared, showed at least four reassortment events with different subtypes (H5N8, H7N7, H3N8, and H10N7) (Kim et al. 2017) (Figure 1). For outbreaks of H5N8 viruses, a total of 89 cases, consisting of 76 cases from farms and 13 cases from wild birds, were reported for 132 days from February to June 2017 (Figure 1 and Table 1 column ‘17).

In 2016, the pathogenicity of H5N6 HPAIV was assessed in two white chicken lines of SPF chickens, broilers, and native chickens (Park et al. 2019) (Table 2). Korean native chickens exhibited prolonged viral shedding and higher survival rates. Experimental pathogenicity studies in broiler ducks revealed that the C-4 genotype virus, isolated from broiler ducks, induced higher mortality compared to the C-1 genotype, isolated from wild mandarin duck (Kwon et al. 2019). This difference in pathogenicity was attributed to reassortment in the PA and NS genes. The pathogenicity and transmissibility of the H5N6 virus ­isolated from a Mandarin duck in 2016 and the H5N8 virus isolated in 2017 were evaluated using Mandarin ducks as experimental models (Son et al. 2018) (Table 3). No mortality or clinical signs were observed in any individuals during the study. Hemagglutination inhibition assays conducted with serum samples ­indicated similar antigenic profiles between the two viruses. Cross-challenge experiments demonstrated effective prevention of reinfection between H5N6 and H5N8 viruses. Interestingly, viral replication and shedding were significantly higher in H5N8-infected individuals compared to those infected with H5N6.

## 8. 2017–2018: Clade 2.3.4.4b H5N6

Following the occurrence of H5N6 avian influenza in wild birds in Chungcheongnam-do and Gyeonggi-do, a farm die-off was reported in November 2017. Genetic analysis confirmed a reassortant virus, comprising clade 2.3.4.4x H5N8 HPAIV and H3N6 LPAIV from the Netherlands (Kim, Si, et al. 2018). In November 2017, active surveillance identified two novel H5N6 HPAIVs: A/duck/Korea/HD1/2017(H5N6) (HD1) from a Gochang duck farm and A/mallard/Korea/Jeju-H24/2017(H5N6) (Jeju-H24) from wild bird feces (Lee et al. 2018). These isolates, similar to HPAIV isolated in whooper swan in Japan, likely resulted from recombination between the NA gene of a 2016 European LPAI virus and the H5N8 HPAIV. For this season, 22 farm cases and 12 wild birds cases were reported for 121 days (Figure 1 and Table 1).

Genetic analysis of H5N6 HPAIVs from duck farms (8 cases) and wild birds (4 cases) between November and December 2017 revealed distinct genotypes (Lee, Cheon, et al. 2018). Using genotyping on the basis of the gene segment-specific phylogenetic tree, with Chinese H5N8 HPAI isolate (2013) and Korean isolates (2014, designated B0), isolates were categorized into B0 to B3 from early 2016 to November 2017. In the latter half of 2017, the B3 lineage diverged into B3.1.1 and B3.2.1 (Figure 1). Whole genome analysis of H5N6 HPAI isolates collected between January and March 2018 identified three distinct genotypes, including a novel genotype, represented by the virus H214, which possesses PB2, PA, and NP genes derived from the Eurasian LPAI gene pool (Baek et al. 2020) (Figure 1). SPF chicken and commercial duck experiments confirmed low pathogenicity in ducks and determined 50% bird lethal dose (BLD_50_) and MDT in chickens (Park et al. 2021) (Table 2).

## 9. 2020–2021: Clade 2.3.4.4b H5N8

From the summer of 2018 to the fall of 2020, no reported outbreaks occurred. However, in October 2020, the clade 2.3.4.4b H5N8 HPAIV was isolated from wild bird feces in Mandarin duck, marking its initial report (Jeong et al. 2020). The genetic analysis revealed that eight segments of the isolated virus were closely related to the H5N8 HPAIV identified in Europe in early 2020. For this period, 337 cases were reported with 103 farm cases and 234 wild bird cases (Figure 1 and Table 1). During the late 2020–2021 winter season, South Korea experienced a large-scale outbreak of H5N8 HPAI, prompting the Ministry of Environment to conduct extensive surveillance in wild birds, resulting in the collection of 7588 samples (Si et al. 2024). These samples comprised 4741 fecal samples, 2400 swab samples from captured live birds, and 477 carcasses. Influenza A viruses were detected in 5.0% (*n* = 384) of the samples, including 263 from fecal samples (5.5%), 9 from swab samples (0.3%), and 112 from carcasses (25.0%). Among these identified viruses, HPAI H5N8 accounted for 38.5% (*n* = 148) of the isolates, with 32 detected in fecal samples (12.1%), 7 in swab samples (77.7%), and 109 in carcasses (97.3%) (Si et al. 2024). Between the first isolation report and January 2021, 67 H5N8 HPAIVs from both wild birds and poultry underwent analysis (Baek et al. 2021). Phylogenetic analysis of the HA gene indicated that all isolates belonged to the H5 clade 2.3.4.4 subgroup B (2.3.4.4b), forming two distinct genetic clusters, G1 and G2. Cluster G1 demonstrated a close relation to the 2.3.4.4b H5N8 HPAIVs detected in Europe in early 2020, while cluster G2 had a genetic relationship with the 2.3.4.4b H5N8 viruses that circulated in Europe in late 2020. Seven distinct genotypes were identified, including five novel reassortants carrying internal genes of LPAI viruses. In wild birds, genetic analysis of viruses revealed that G1 and G2 viruses were introduced separately into Korea. Similar to poultry, G1 genotype viruses gave rise to various sub-genotypes (G1-1 to G1-5), predominantly isolated from clinical specimens, while the G2 genotype viruses were introduced later and were primarily detected in dead wild birds (Figure 1) (Si et al. 2024).

Reports on the pathogenicity and transmission in SPF chickens and commercial ducks using the first isolated strain, the H242/20 (H5N8), in 2020 were documented (Park et al. 2021) (Table 2). In chickens, the 50% chicken lethal dose (cLD50) and MDT for the H242/20 (H5N8) strain were 10^4.5^ EID_50_ and 4.3 days, respectively, indicating lower virulence compared to those of the HD1/17 (H5N6) strain (10^3.6^ EID_50_ and 2.2 days), circulated in 2017 in South Korea (Table 2). Chickens inoculated with H242/20 (H5N8) survived longer and exhibited higher viral shedding titers, potentially increasing farm contamination risk. Ducks infected with either HPAIV showed no clinical symptoms, but had prolonged virus shedding and higher transmission rates, suggesting their role as silent carriers.

## 10. 2021–2022, 2022–2023, 2023–2024: Clade 2.3.4.4b H5N1, H5N6

In the latter of 2021, the clade 2.3.4.4b H5N1 HPAIV was isolated and subjected to genetic analysis, confirming its status as a novel reassortant (Sagong et al. 2022). Genetic analysis of 47 H5N1 HPAIVs from farm outbreaks during the 2021–2022 season revealed similarities to viruses primarily found in Eurasian regions, identifying four distinct genotypes (Figure 1). These viruses were prevalent not only in poultry but also in wild birds. For the winter season of 2021–2022, 114 cases (47 farm cases and 67 wild bird cases) were reported (Table 1). A representative H5N1 strain exhibited high pathogenicity and efficient transmission, particularly in chickens and ducks during the spring (Cha et al. 2023) (Table 2).

In October 2022, the same subtype of HPAIVs were isolated from wild birds, including Mandarin duck and common teal, as well as from domestic breeder duck farms (Kang et al. 2023). It was confirmed that at least two genotypes of HPAIVs were introduced, and viruses bearing similar constellations of one of these genotypes were also detected in China, Russia, and Korea. The other genotype virus containing four genes (PB2, PB1, NP, and NS) originating from LPAI virus isolated in North America, suggesting viral spread through intercontinental bird migration. Based on Genoflu analysis, this virus was identified as the unreassorted B3.2 genotype, which was previously detected in Alaska and corresponds to one of the H5N1 genotypes that circulated in the United States from late 2021 to 2022 (Youk et al. 2023). In November 2022, the clade 2.3.4.4b H5N1 virus was isolated from Spot-billed duck feces, with genetic analysis revealing the presence of two novel reassortants among the five viruses (Lee et al. 2023). Between October 2022 and March 2023, 75 cases were reported from poultry farms and 174 cases of HPAI infections were confirmed in wild birds (Table 1). Among HPAIVs isolated from wild birds, there are 16 distinct genotypes (Kor22-23A-P) (Figure 1) through genetic analysis of 113 complete HPAI genomes (Seo, Cho, et al. 2024). Notably, during this period, from November to December 2022, a significant die-off of 221 hooded cranes (*Grus monacha*) was reported in Suncheon Bay. The viruses isolated from these cranes were identified as clade 2.3.4.4b H5N1 HPAIVs, specifically belonging to the Kor22-23C and Kor22-23B genotypes (Seo, Lee, et al. 2024). Additionally, between June 24 and 27, 2023, three out of 40 cats at a non-profit private shelter died after showing high fever and anorexia, and genetic analysis of the HPAIVs ­isolated from these cats revealed that the HA, NA, and M genes were closely related to the G10 genotype previously reported in China and Korea, while the remaining genes originated from Eurasian LPAI viruses (Lee et al. 2024).

In November 2023, a H5N1 HPAIV was isolated from a Eurasian wigeon, clustering with clade 2.3.4.4b H5N1 viruses detected in Japan during 2022–2023, without evidence of reassortment (Cho, Si, Lee, et al. 2024). Following the first report, H5N6 HPAIVs were isolated from carcasses of whooper swans and bean geese (Cho, Si, Kim, et al. 2024), showing reassortment events with the PB1, HA, and M genes of the 2022–2023 H5N1 HPAIV strains and the NA gene from H5N6 HPAIV strains identified in China in 2021, with the remaining genes derived from LPAI viruses. By December 2023, concurrent outbreaks of H5N6 and H5N1 were reported in poultry (Heo et al. 2024), with the H5N1 strains displaying a complete HPAI-derived constellation without reassortment. In the H5N6 strains, the PB1, HA, and M genes originated from Korean and Japanese HPAI strains, while the other genes were derived from LPAIV (Figure 1). In total, 51 cases were recorded, with 32 cases in poultry and 19 in wild birds (Table 1).

In animal experiments using HPAIV isolated during 2021-2022, the WA585/21 H5N1 HPAIV, isolated from wild birds in this season, demonstrated high pathogenicity and efficient transmission in chickens (Cha et al. 2023) (Table 2). Conversely, ducks infected with the virus displayed no mortality but showed higher rates of transmission and longer viral shedding compared to chickens, suggesting their potential role as silent carriers.

## 11. Concluding remarks

The timing of outbreaks has most frequently aligned with the arrival of wintering migratory birds, peaking between October and December. Notably, exceptions occurred in 2008, 2014–2016, and 2017, when outbreaks emerged during the first half of the year. Since the 2017–2018 season, outbreaks have consistently begun in October or November. The outbreak duration varied significantly, with the longest periods recorded during the 2014–2016 seasons, while 2008 marked the shortest at just 42 days. Most other outbreaks persisted for 100–200 days. Crucially, advancements in surveillance systems and farm biosecurity measures, coupled with efforts to block virus introduction through migratory birds, appear instrumental in mitigating the spread of HPAI, breaking the previous trend of protracted outbreaks with limited control.

Before the 2017–2018 season, most cases were reported on poultry farms, whereas wild bird cases became more prevalent in the subsequent three years. Case numbers rose steadily until the 2014–2016 seasons, declined by the 2017–2018 season, and have since fluctuated. Poultry farm outbreaks primarily affected chickens and ducks, with ducks experiencing the highest case numbers during the 2015–2016 season and chickens peaking in 2016–2017. The gap in case numbers between the two species farms has since narrowed, with fewer than seven cases after the 2017–2018 season. This trend reflects the effectiveness of active surveillance, early detection, culling, and biosecurity measures. In wild birds, most detections came from carcasses, which may be attributed to sampling limitations, and in some seasons, viruses introduced into wild bird populations caused significant mortality, leading to large numbers of detections. Since the 2020–2021 season, large-scale mortality events have occurred biennially, suggesting potential factors such as the endemic nature of HPAIVs, pre-existing immunity from previous infections, and characteristics of new virus strains as contributing to this pattern.

Before the 2010–2011 season, a single genotype of the virus was predominantly introduced each season, however, since 2014, the emergence of reassortant viruses involving segments from LPAI viruses has significantly increased genotypic diversity. Notably, after the detection of viruses with all eight genome segments closely related with a specific HPAIV, new genotypes resulting from reassortment were identified during the 2020–2021 and 2022–2023 seasons. This trend suggests a heightened potential for the emergence of viruses with novel characteristics, underscoring the critical need for comprehensive analysis of the diverse genotypes to better understand their implications and mitigate future risks.

Pathogenicity studies showed that SPF chickens exhibited cLD_50_ values ranging from 10^3.0^ to 10^5.3^ EID_50_, with varying MDTs between 2.2 and 4.3 days. The virus with the highest sensitivity and lethality in chickens was the 2006–2007 strain, with cLD_50_ of 10^2.3^EID_50_. The virus causing the fastest mortality was the 2017–2018 strain, exhibiting an MDT of 2.2 days. Based on cLD_50_ values, virus lethality was relatively lower in the 2014–2016 and 2020–2021 seasons. Except for the season with the lowest value, most viruses displayed cLD_50_ values close to 10^3^ EID_50_. Notably, the 2020–2021 virus had the longest MDT at 4.3 days, potentially contributing to the observed increase in cases on poultry farms by delaying mortality. In ducks, BLD_50_ values were only observed in viruses isolated before 2011 and a single strain from the 2016–2017 season, suggesting generally lower pathogenicity in this species. In ducks, 50% bird infectious dose (BID_50_) values generally ranged from 10^3.0^ to 10^5.3^ EID_50_. After the 2016–2017 season, duck BID_50_ values exceeded 10^5.0^ EID_50_ for two consecutive years, indicating reduced sensitivity. However, in the subsequent year, duck BID_50_ values decreased, suggesting a recovery in sensitivity.

For wild birds, assessing pathogenicity has been challenging due to the lack of consistent species, age, and testing protocols, though it has been found that, aside from mortality in swans and ruddy shelducks in the 2003–2004 season, most wild birds did not show mortality but shed the virus, with some species showing transmission while others did not. This suggests that certain wild bird species may play a primary role in virus transmission, and further evaluation of species-specific pathogenicity and transmissibility for each season’s representative strains is necessary.

In contemporary landscape, the Gs/GD H5 sub-lineages of the HPAIV are undergoing rapid evolution, emerging as the primary instigators of infectious outbreaks in poultry. Our comprehensive review delves into the occurrence patterns, cases and affected species, along with exploring the pathogenicity in animals and genetic characteristics of HPAI continuously introduced in Korea. With the enhancement of biosecurity measures compared to previous years, there has been a notable reduction in farm outbreaks. Anticipated is an increased detection rate in wild birds as surveillance intensifies, potentially influencing the overall occurrence dynamics. From outbreaks of varying scales to unprecedented occurrences with potential wild bird mortality and extensive impacts on poultry holdings, each novel outbreak has unveiled a distinctive array of genetic and epidemiological features.

These collective challenges underscore the imperative need for continuous virus monitoring, surveillance of both poultry and wild birds, and comprehensive characterization and pathogenicity assessment of these viruses across diverse animal models. The pertinent information derived from such efforts should be readily available ahead of any potential outbreak in poultry and wild birds. Also, timely genetic analysis of viruses isolated from poultry and wild birds each season is important for sharing this information with neighboring countries and fostering collaborative preparedness. This proactive approach enables the formulation of specific preparation and containment strategies, particularly in countries designated as ‘sink’ regions, thereby mitigating the risk of further epidemics. The imminent introduction of novel HPAI H5 viruses on a global scale underscores the importance of these preparatory measures in averting potential future crises.

## Supplementary Material

Supplemental Material

## Data Availability

The datasets analyzed for this review are available upon request from the corresponding author. Requests to access the datasets should be directed to Chang-Seon Song at songcs@konkuk.ac.kr.
