# Supplementary material for: Epidemiology and pathobiology of H5Nx highly pathogenic avian influenza in South Korea (2003–2024): a comprehensive review
Source: Vet Q. 2025 May 7;45(1):23–38. doi: 10.1080/01652176.2025.2498918 (PMC12064103; doi:10.1080/01652176.2025.2498918)
Supplement: Supplemental Material [file TVEQ_A_2498918_SM8814.pdf]

Supplementary Table 1. Experimental infections in various species of mammalian animals with HPAIVs isolated in South Korea from 2003 to 2022.

period	H5 Clade[a]	sub-type	strain	animal	pathogenicity index	contents	results	reference
’03-04	2.5	H5N1	A/chicken/Korea/ES/2003	ferret		pathogenicity<br>body weight<br>temperature	Mortality[e] (0/3)<br>Viral shedding[f] NW (3/3)	Kim et al. 2015
’06-07	2.2	H5N1	A/EM/Korea/W149/06	Mice,	MLD_{50}[b]$=10^{3.3}EID_{50}$[c]	pathogenicity<br>transmission<br>viral shedding<br>tissue viral titers<br>transmission in ferret	Mortality (6/6)<br>Tissue distribution[f] (brain, kidney, spleen, intestinal tract, heart)	Kwon et al. 2011
				Ferret			Mortality (0/3)<br>Viral shedding NW (3/3)<br>Transmission (0/2)<br>Tissue distribution (lung, kidney, spleen)	
			A/environment/Korea/W149/2006	Mice	$MLD_{50}=10^{3.3}EID_{50}$	pathogenicity<br>tissue viral titers	Mortality (10/10)<br>Tissue distribution (lung, brain, kidney, spleen, heart, liver, colon)	Kim et al. 2014
				Ferret		pathogenicity<br>viral shedding<br>tissue viral titers<br>transmission	Mortality (0/3)<br>Viral shedding NW (3/3)<br>Tissue distribution (lung, kidney, spleen)<br>Transmission (0/3)	
‘08	2.3.2	H5N1	Ck/Kr/Gimje/08	Mice	$MID_{50}=10^{1.5}EID_{50}$<br>$MLD_{50}=10^{2.2}EID_{50}$	pathogenicity<br>tissue viral titer<br>viral shedding	Mortality (5/5)<br>Tissue distribution (brain, lung, liver, spleen, kidney, heart)	Kang et al. 2013
’10-11	2.3.2	H5N1	A/Md/Korea/W401/11	Mice	$MLD_{50}=10^{3.9}EID_{50}$	pathogenicity<br>transmission<br>viral shedding<br>tissue viral titers<br>transmission in ferret	Mortality (6/6)<br>Tissue distribution (brain, kidney, spleen, intestinal tract, heart)	Kim et al. 2014
				Ferret			Mortality (0/3)<br>Viral shedding NW (3/3)<br>Transmission (0/2)<br>Tissue distribution (lung)	
’10-11	2.3.2.1c	H5N1	A/mandarin duck/Korea/PSC24-24/2010	Mice	MID_{50}[d]$=10^{2.3}EID_{50}$<br>$MLD_{50}=10^{3.4}EID_{50}$	viral shedding<br>body weight<br>survival curve<br>transmission<br>tissue viral titer	Mortality (5/5)<br>Tissue distribution (lung, spleen, brain)	Choi et al. 2013
’10-11	2.3.2.1c	H5N1	A/Eurasian eagle owl/Korea/23/2010	Mice	$MID_{50}=10^{1.5}EID_{50}$<br>$MLD_{50}=10^{3.6}EID_{50}$	viral shedding<br>body weight<br>survival curve<br>transmission<br>tissue viral titer	Mortality (5/5)<br>Tissue distribution (lung, spleen, brain)	Choi et al. 2013
’14-16	2.3.4.4c	H5N8	A/mallard duck/Korea/W452/2014	Mice	$MLD_{50}=10^{5.5}EID_{50}$	pathogenicity<br>transmission<br>viral shedding<br>tissue viral titers<br>transmission in ferret	Mortality (4/10)<br>Tissue distribution (Lung, brain)	Kim et al. 2014
				Ferret			Mortality (0/3)<br>Viral shedding NW (3/3)<br>Tissue distribution (lung, brain, spleen, liver, colon)<br>Transmission (1/3)	
				Dog			Mortality (0/2)<br>transmission (0/3)<br>Viral shedding NW (3/7)<br>Tissue distribution (Lung)	
				Cat			Mortality (0/2)<br>Viral shedding NS (3/3)<br>Tissue distribution (Nasal turbinate, trachea, Lung)<br>transmission (1/3)	

'14-16	2.3.4.4b	H5N8	A/broiler duck/korea/Buan2/2014	Ferret		pathogenicity<br>body weight<br>temperature<br>viral shedding	(i.n.) Mortality (0/8)<br>(i.n.) Viral shedding NW (8/8)	(i.t.) Mortality (8/8)	Kim et al. 2015
	2.3.4.4c		A/breeder duck/korea/Gochang1/2014				(i.n.) Mortality (0/8)	(i.n.) Viral shedding NW (8/8)	
			A/Baikal teal/Korea/K14-E016/2014	Dog		transmission<br>viral shedding<br>pathogenicity	Viral shedding NS (2/4)<br>Contact transmission (1/4)		Yuk et al. 2017
			A/broiler duck/Korea/H1731/2014	Mice	$MID_{50}=10^{4.5}EID_{50}$<br>$MLD_{50}=10^{6.5}EID_{50}$	pathogenicity<br>tissue viral titer	Mortality (1/5)	Tissue distribution (lung, spleen)	Lee et al. 2018
				Ferret			Viral shedding NW (6/6)	Tissue distribution (nasal turbinate, lung)	
			A/domestic mallard_duck/Korea/H1924/2014	Mice	$MID_{50}=10^{5.5}EID_{50}$<br>$MLD_{50}>10^{7.0}EID_{50}$		Mortality (0/5)	Tissue distribution (lung, spleen)	
				Ferret			Viral shedding NW (6/6)	Tissue distribution (trachea)	
			A/mallard duck/Korea/H2102/2015	Mice	$MID_{50}=10^{4.2}EID_{50}$<br>$MLE_{50}=10^{5.3}EID_{50}$		Mortality (4/5)	Tissue distribution (lung, spleen)	
				Ferret			Viral shedding NW (6/6)	Tissue distribution (nasal turbinate, lung)	

[a] Lineage of virus used in each study was replaced with updated unified nomenclature made by WOAH.
[b] MLD_{50}; 50% mouse lethal dose
[c] EID_{50}; 50% egg infectious dose
[d] MID_{50}; 50% mouse infectious dose
[e] Transmission, mortality based on highest viral challenge groups. Transmission was confirmed based on viral quantification or seroconversion.
[f] Virus shedding and tissue distribution deduced by viral detection. Virus shedding only stated for inoculated animals. NW; nasal wash, NS; nasal swab
